# A Multicenter Retrospective Study Evaluating IL-5-Targeted Biologic Therapies for the Treatment of Asthma and Allergic Bronchopulmonary Aspergillosis in Adults With Cystic Fibrosis

**DOI:** 10.1016/j.chpulm.2025.100163

**Published:** 2025-03-12

**Authors:** Sameer Desai, Sophia Shen, Zosia Gryz, Elizabeth Tullis, Anne L. Stephenson, Bradley S. Quon

**Affiliations:** a School of Population and Public Health, University of British Columbia, Vancouver, BC, Canada; b Department of Biostatistics, Epidemiology, and Scientific Computing, King Faisal Specialist Hospital and Research Centre, Riyadh, Saudi Arabia; c Faculty of Medicine, University of British Columbia, Vancouver, BC, Canada; d Division of Respirology, St Michael’s Hospital, University of Toronto, Toronto, ON, Canada; e Division of Respiratory Medicine, Department of Medicine, University of British Columbia, Vancouver, BC, Canada; f Centre for Heart Lung Innovation, St Paul’s Hospital and the University of British Columbia, Vancouver, BC, Canada

**Keywords:** allergic bronchopulmonary aspergillosis, asthma, benralizumab, cystic fibrosis, mepolizumab, ABPA, Th2

## Abstract

**Background:**

Biologic therapies targeting T-helper cell type 2 (Th2) eosinophilic inflammation have been used for difficult-to-treat asthma and allergic bronchopulmonary aspergillosis (ABPA), but safety and efficacy data are limited in the cystic fibrosis (CF) population.

**Research Question:**

Are Th2 biologic therapies targeting eosinophilic inflammation effective and safe when used in adults with CF with asthma and/or ABPA overlap?

**Study Design and Methods:**

This was a retrospective multicenter study. Individuals with CF receiving Th2 biologic therapies for asthma or ABPA at 2 large adult CF centers between 2016 and 2021 were included. Treatment safety and effectiveness up to 12 months after biologic initiation were evaluated. Outcomes of interest included percent predicted FEV_1_ (ppFEV_1_), rate of change in ppFEV_1_, cumulative systemic corticosteroid (SCS) dose, and frequency of pulmonary exacerbations (PExs).

**Results:**

Forty adults with CF received Th2 biologic therapies targeting eosinophilic inflammation during the study period. A total of 38 were eligible for study inclusion, and all received IL-5-directed therapies (mepolizumab: n = 27, benralizumab: n = 11): 17 (45%) for asthma only and 21 for ABPA (55%) with or without asthma. No significant biologic-related adverse effects were observed. There was no statistical difference in ppFEV_1_ from baseline to 12-months postbiologic initiation, but the rate of change in ppFEV_1_ improved in the 12 months postbiologic vs prebiologic with a pre-to-post difference of 3.7% (95% CI, 1.2%-6.3%). SCS dose and frequency of PExs requiring hospitalization did not differ significantly in the 12 months before biologic vs after biologic initiation.

**Interpretation:**

Our results show that in adults with CF, anti-IL-5 biologic therapies were well tolerated and can improve rate of change in lung function, but there was no impact on SCS use or PEx frequency. Further study is needed to better define the role of Th2 biologics in the era of highly effective modulator therapy.

**Take-Home Points**

**Study Question:** Are T-helper cell type 2 biologic therapies effective and safe when used in adults with cystic fibrosis with asthma and/or allergic bronchopulmonary aspergillosis overlap?

**Results:** In the first year after initiation of an IL-5-targeted biologic (mepolizumab, benralizumab), the rate of decline in lung function decreased, but the need for systemic corticosteroids and frequency of pulmonary exacerbations requiring hospitalization did not change.

**Interpretation:** Our results indicate that IL-5-targeted biologics are safe in adults with cystic fibrosis but lead to variable clinical responses, and their role in the era of highly effective cystic fibrosis transmembrane conductance regulator modulators treatments remains uncertain.

People with cystic fibrosis (pwCF) are at increased risk of developing asthma and allergic bronchopulmonary aspergillosis (ABPA),^1^ allergic airway conditions mediated by a T-helper cell type 2 (Th2) adaptive immune response and characterized by eosinophilic inflammation. ABPA develops due to sensitization to allergens from the fungus *Aspergillus fumigatus*, which is ubiquitous in the environment and commonly found in the sputum of pwCF. It has been estimated that approximately 5% of pwCF are diagnosed with ABPA and approximately 30% with asthma at some point during their lifetime.^1^

The mainstay of therapy for asthma and ABPA exacerbations are systemic corticosteroid (SCSs).^2^^,^^3^ In addition to the typical side effects associated with SCSs,^4^ there can be additional adverse consequences in the cystic fibrosis (CF) population specifically due to immunosuppression, with the potential to exacerbate airway infection. Furthermore, comorbid conditions (eg, diabetes, bone demineralization) are common in CF and can be worsened by SCS use.^5^ Omalizumab, a monoclonal IgE antibody, has been used off-label as a steroid-sparing agent to target allergic airway inflammation (asthma and ABPA) in CF but with mixed results.^6^, ^7^, ^8^, ^9^, ^10^, ^11^

Over the last decade, a number of new steroid-sparing Th2 biologic agents have been developed to target eosinophilic inflammation, including humanized monoclonal antibodies mepolizumab,^12^ benralizumab,^13^ reslizumab,^14^ dupilumab,^15^ and tezepelumab.^16^ These biologic agents have been approved for use in severe allergic/eosinophilic asthma and target specific molecules from the Th2 pathway. Data supporting the use of Th2 biologics in pwCF are scarce. To date and to our knowledge, only 1 case report and a small case series involving 3 pwCF have reported on the effectiveness and safety of Th2 biologics for the treatment of ABPA.^17^^,^^18^ The case report documented mepolizumab use in an adult with CF, which resulted in symptomatic and radiographic improvement.^17^ In the case series, mepolizumab was well tolerated, and there was a reduction in the requirement for oral corticosteroids, but there was no change in the frequency of exacerbations.^18^

The goal of this retrospective multicenter observational study was to evaluate the effectiveness and safety of Th2 biologic therapies for asthma and ABPA in the adult CF population.

## Study Design and Methods

This was a retrospective observational study including adults with CF who initiated and completed at least 3 months of Th2 biologic therapy targeting eosinophilic inflammation for uncontrolled asthma and/or ABPA at 2 large adult CF clinics (St Paul’s Hospital, Vancouver, BC, Canada; St Michael’s Hospital, Toronto, ON, Canada) between August 15, 2016, and January 15, 2021. Th2 biologics of interest included those approved by Health Canada for severe eosinophilic asthma during the study period including mepolizumab, reslizumab, benralizumab, and dupilumab. The Th2 biologic omalizumab was not included because we were interested in examining the effects of Th2 biologic therapy targeting eosinophilic inflammation more specifically. PwCF on Th2 biologics of interest were identified via chart review, and their clinical data were retrieved from clinical charts and via linkage to the Canadian Cystic Fibrosis Registry. All individuals included in the Canadian Cystic Fibrosis Registry provided their written informed consent to have their data linked for research purposes. The study protocol was approved by the ethics committees at the respective institutions (PHC REB H20-01845; SMH REB 21-017).

Included individuals had a diagnosis of asthma and/or ABPA verified by at least 2 CF physicians from each site and guided by CF diagnostic criteria.^2^^,^^3^ Treatment effectiveness and safety were assessed by evaluating outcomes up to 12 months after biologic treatment. To evaluate the effect on lung function, the closest percent predicted FEV_1_ (ppFEV_1_) value obtained just before biologic initiation was compared with the closest ppFEV_1_ value obtained 12 ± 3 months after biologic treatment. A patient’s clinical status at the time of lung function assessment might affect the results. To address this, we conducted a sensitivity analysis using the median of all ppFEV_1_ values obtained 12 months before biologic initiation to represent the baseline ppFEV_1_ (instead of the last ppFEV_1_ measurement just before biologic initiation) and 12 months after biologic initiation. Additionally, rate of change in ppFEV_1_ was calculated by using all ppFEV_1_ measurements obtained in the 12 months before biologics vs after biologics. e-Figure 1 shows the flowchart for the creation of the analytical cohorts. The steroid-sparing effect of Th2 biologic treatments was assessed by comparing changes in the cumulative dose of SCSs (expressed as the equivalent of prednisone dose) and the proportion of individuals who had a 50% decrease in SCS dose in the 12 months prebiologic vs postbiologic. Frequency of pulmonary exacerbation (PEx) requiring hospitalization per patient and number of days requiring hospitalization were examined 12 months before biologics vs after biologics. Regarding treatment safety, any adverse effects attributable to Th2 biologics according to physician assessment based on chart review and reasons for treatment discontinuation up to 12 months after biologics were recorded.

We also performed exploratory subgroup analyses to evaluate treatment response based on treatment indication and biomarkers of Th2 inflammation. For treatment indication, patients were divided into 2 groups: asthma only and ABPA with or without asthma. For subgroup analyses evaluating absolute eosinophil count (AEC) (cells/mL) as a biomarker of Th2 inflammation, cutoff AECs of ≥ 400 cells/μL or < 400 cells/μL and ≥ 500 cells/μL or < 500 cells/μL were explored because all individuals had to have an AEC > 300 cells/μL to be eligible for Th2 biologic reimbursement.

### Statistical Analysis

Demographics and baseline clinical characteristics of this cohort were described using simple descriptive statistics (eg, means, medians, proportions). A nonparametric paired Wilcoxon signed-rank test was used to compare the average ppFEV_1_ change from before biologics to 12 months after biologics treatment. Only patients with complete data (minimum 9 months of follow-up) were included in this analysis. For the change in rate of ppFEV_1_ over time, a segmented generalized linear random effects model was used. This analysis considered all ppFEV_1_ measurements taken 12 months before biologics and up to 12 months after biologics, using the date of biologic initiation as time zero. The main parameter of interest was the change in ppFEV_1_ slope before biologic vs after biologic initiation. Random intercepts for within-patient variation were treated as random effects in the model with an unstructured covariance pattern. Cumulative SCS use 12 months before biologics and after biologics was compared with the Wilcoxon signed-rank test. PEx frequency 12 months before biologics and after biologics was compared with a generalized linear random effects model. The model assumed a Poisson distribution and incorporated an offset term to accommodate varying observation times for each patient. We calculated incidence rate ratio to assess PEx counts 12 months before biologic and 12 months after biologic initiation. Similar to the ppFEV_1_ analysis, random intercepts were included in the model, and an unstructured covariance pattern was specified. Individuals started on a cystic fibrosis transmembrane conductance regulator (CFTR) modulator therapy post-Th2 biologic initiation were censored or excluded (if applicable) as part of the sensitivity analysis.

The level of significance was set at *P* < .05 for all statistical analyses, and all reported *P* values reflect 2-tailed tests. All analyses were conducted using R (version 4.3.1; The R Foundation for Statistical Computing). Statistical modeling was performed using the lme4 package,^19^ and summary tables were generated using the gtsummary package.^20^

## Results

Forty adults with CF (30 from St Michael’s Hospital, 10 from St Paul’s Hospital) received Th2 biologic treatments during the study period. One individual was excluded because the date of biologic initiation could not be ascertained and 1 individual did not have data after biologic initiation. Of 38 individuals included in this analysis, 27 received mepolizumab and 11 received benralizumab. No individuals during the study period received reslizumab, dupilumab, or tezepelumab. Sixteen individuals (42%) had previously received omalizumab before initiating a Th2 biologic. Of these, 7 discontinued omalizumab within 2 months before initiating the Th2 biologic, whereas 5 individuals (13%) continued omalizumab after starting the Th2 biologic. Seventeen individuals (45%) had a diagnosis of asthma only, and 21 (55%) had a diagnosis of ABPA with or without asthma.

Median age at time of biologic initiation was 35 years (interquartile range [IQR], 26-47), with a similar proportion of male and female participants. Most individuals had moderate obstructive lung disease with a median ppFEV_1_ of 50% (IQR, 40%-63%), and most participants had chronic *Pseudomonas aeruginosa* and *Aspergillus* species infections (Table 1). Before initiation of a Th2 biologic, 6 individuals (16%) were on a CFTR modulator (lumacaftor-ivacaftor: n = 4, tezacaftor-ivacaftor: n = 2) at least 3 months before biologic initiation. The clinical characteristics of the ABPA and asthma only subgroups were not significantly different, but there was a trend toward a higher proportion of males, *P* *aeruginosa* infection, *Aspergillus*-specific IgE positivity, and higher IgE concentrations in those with ABPA vs asthma only (e-Table 1).

**Table 1:** Clinical Characteristics of Patients With CF Receiving T-Helper Cell Type 2 Biologics (N = 38)

Characteristic	Value
Age at biologic initiation, y	35 (26-47)
Hospital	
St Michael’s Hospital (Toronto, ON, Canada)	28 (74)
St Paul’s Hospital (Vancouver, BC, Canada)	10 (26)
Female sex	19 (50)
CF genotype	
Homozygous F508del	18 (47)
Heterozygous F508del	14 (37)
Other	6 (16)
Diagnosis	
Asthma alone	17 (45)
ABPA (with or without asthma)	21 (55)
Sputum microbiology PA positive	30 (79)
Sputum microbiology *Aspergillus* positive	22 (58)
ppFEV_1_ at biologic initiation	50 (40-63)
BMI, kg/m^2^	22.8 (20.3-25.6)
Pancreatic insufficient	33 (87)
CFRD	25 (66)
Total IgE, kU/L	179 (49-298)
*Aspergillus* precipitins positive (n = 23)	8 (35)
Elevated *Aspergillus*-specific IgE (n = 20)^a^	14 (70)
Absolute eosinophil count, cells/μL	540 (360-790)
Absolute eosinophil count ≥ 500 cells/μL (n = 23)^b^	11 (48)
Prior use of omalizumab (within 1 y before biologic initiation)	11 (29)
Concurrent use of omalizumab (at the time of biologic initiation or postbiologic initiation)	5 (13)
Systemic corticosteroid use before biologic initiation	29 (76)
Daily prednisone dose, mg/d	7.0 (0.5-16.7)
CFTR modulator use before biologic^c^	6 (16)
Follow-up time postbiologic, mo^d^	13.0 (10.0-13.7)

Values are presented as median (interquartile range) or No. (%). ABPA = allergic bronchopulmonary aspergillosis; CF = cystic fibrosis; CFRD = CF-related diabetes; CFTR = cystic fibrosis transmembrane conductance regulator; kU/L=kilo units per litre; PA = *Pseudomonas aeruginosa*; ppFEV_1_ = percent predicted FEV_1_.

a Cutoff > 0.35 International Units/mL.

b Only for patients not on prednisone.

c Four of 6 discontinued before biologic initiation.

d From FEV_1_ measurement just before biologic start to last visit closest to 12 mo after biologic initiation.

In terms of biomarkers of Th2 inflammation, the median serum IgE level and blood eosinophil count at baseline were 179 kU/L (IQR, 49-298) and 540 cells/μL (IQR, 360-780), respectively. For individuals not on SCSs (n = 23) at baseline, 17 (74%) had a blood AEC of ≥ 400 cells/μL and 11 (48%) had a blood AEC of ≥ 500 cells/μL. Of the 20 individuals with *Aspergillus*-specific IgE levels measured, 14 (70%) were elevated based on a cutoff value of ≥ 0.35 International Units/mL.

**Figure 1:**
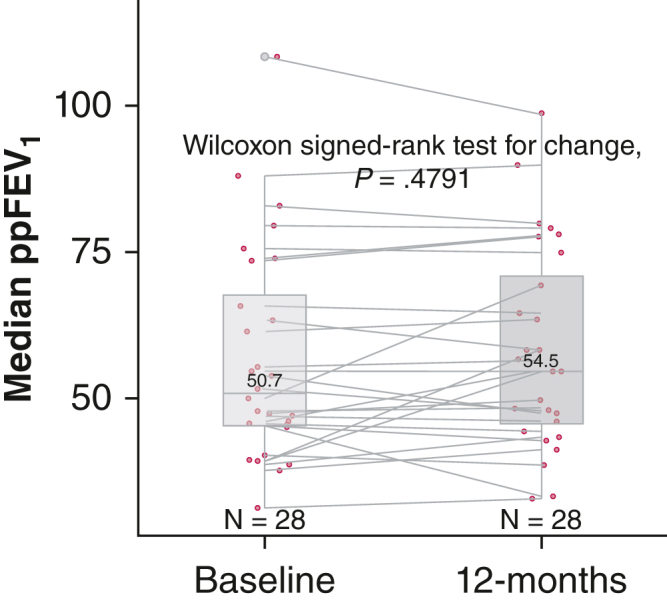
Change in average ppFEV_1_ from baseline to 12-mo after biologic initiation. ppFEV_1_ = percent predicted FEV_1_.

Twenty-eight individuals completed 12 ± 3 months of Th2 biologic treatment. Three individuals discontinued treatment before 9 months and 7 people remained on treatment but either were on therapy for < 9 months (n = 3) or had missing outcome data (n = 4). Based on a median follow-up of 13.0 months (IQR, 10.0-13.7), the median ppFEV_1_ increased from 50.7 to 54.5, but the change was not statistically significant (*P* = .48) (Fig 1). Comparison of the median ppFEV_1_ before initiating Th2 biologic with the median ppFEV_1_ after treatment did not alter the findings (e-Figs 2, 3). Post hoc analyses removing 1 individual who started a CFTR modulator after biologics and individuals concurrently using omalizumab (n = 5) also did not alter the primary results (e-Figs 4, 5). An exploratory analysis of the clinical characteristics of responders (defined as ≥ 3% improvement in ppFEV_1_)^21^ and nonresponders revealed no notable trends or significant differences between the 2 groups (e-Table 2).

**Figure 2:**
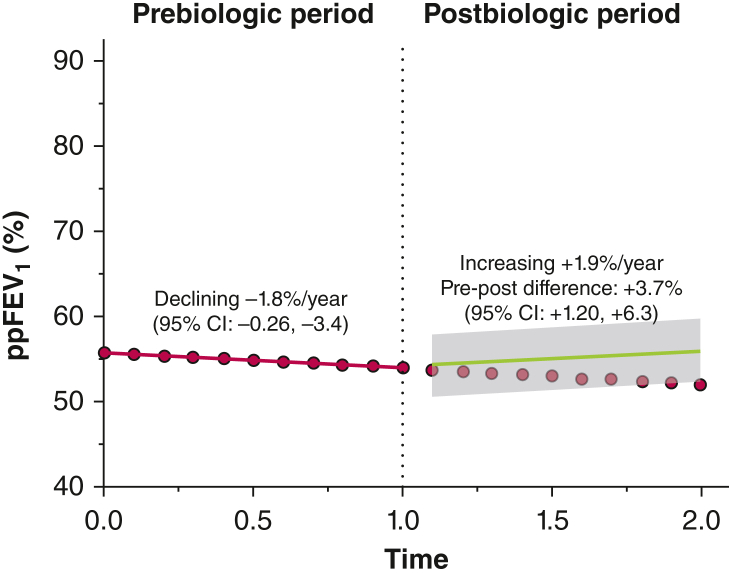
Segmented generalized linear random effect model examining change in ppFEV_1_ over time. All ppFEV_1_ measurements taken 12 mo before and after biologics are included. Lines are model-predicted trends of ppFEV_1_ over time with 95% confidence bands using bootstrapping (n = 1,000). Red dotted line is the postbiologic ppFEV_1_ rate if prebiologic trends continued. Green solid line is the model-predicted trend based on observed data from the postbiologic period. ppFEV_1_ = percent predicted FEV_1_.

Based on segmented regression analysis (n = 38), ppFEV_1_ changed at a rate of −1.8% per year before Th2 biologic and 1.9% per year after biologics, with an immediate effect of 0.1% (95% CI, −1.4% to 1.7%) and a pre-to-post difference of 3.7% (95% CI, 1.2%-6.3%) (Fig 2). Sensitivity analyses were performed evaluating the impact of censoring individuals (n = 3) who started on CFTR modulators after Th2 biologics and also excluding individuals concurrently using omalizumab (n = 5), and the results were also similar (e-Figs 5, 6). Furthermore, the addition of sex, baseline ppFEV_1_, and SCS use as covariates did not alter the findings from the model (e-Table 3).

Cumulative SCS dose decreased 12 months prebiologic to 12 months post-Th2 biologic, but the decrease was not statistically significant (paired median difference, −559 mg; 95% CI, −1,552 to 369; *P* = .25). Because follow-up time was not identical for all individuals both before biologics and after Th2 biologics, the prednisone dose was standardized based on follow-up time and expressed as an average daily dose. The median daily dose decreased from 7 to 3.9 mg/d, but again the decrease was not statistically significant (*P* = .67) (e-Fig 7). The individual changes were heterogeneous, with 29% experiencing a decrease in cumulative SCS dose by 50% and 26% experiencing an increase in dose by 50%.

The mean annual number of PExs per patient requiring hospitalization decreased from 1.43 in the year before therapy to 1.11 in the year after Th2 biologic therapy, but the decrease was not statistically significant with an incidence rate ratio of 0.76 (95% CI, 0.52-1.18; *P* = .20). There was no significant change in the proportion of individuals with at least PEx requiring hospitalization in the year before biologic vs after biologic therapy (65% vs 53%, respectively; *P* = .30). The mean annual number of days per patient in hospital decreased from 24.9 to 18.3, but the decrease also was not statistically significant.

In subgroup analyses, the median increase in ppFEV_1_ from baseline to 12 months after biologics (e-Fig 8) and the mean decrease in the frequency of PEx requiring hospitalization 12 months after biologics (vs 12 months before biologics) (e-Fig 9) were marginally greater in the high (vs low) blood AEC subgroups, but the differences were not statistically significant. There were no significant differences in the change in daily SCS dose after biologics between AEC subgroups (e-Fig 10). There was no difference in the change in ppFEV_1_ from baseline to 12 months after biologics in the asthma (vs ABPA) subgroups (e-Fig 11). Based on the trend lines from the spaghetti plots illustrating the individual changes in ppFEV_1_ before biologics and after biologics, the ABPA/asthma group declined more rapidly before biologics and then stabilized, whereas the asthma-only group remained stable throughout (e-Fig 12). The mean decrease in the frequency of PExs requiring hospitalization 12 months after biologics (vs 12 months before biologics) (e-Fig 13) and in the median decrease in daily SCS dose (e-Fig 14) in the 12 months after biologics (vs 12 months before biologics) were greater in the asthma (vs ABPA) subgroups, but the differences were not statistically significant.

Two individuals reported adverse events associated with mepolizumab. One individual frequently experienced myalgias after injections and 1 individual experienced chest pain. No adverse events were reported for benralizumab. Within 1 year of follow-up, no individuals stopped Th2 biologic therapy due to adverse events, and no patients died or required lung transplant.

## Discussion

In this retrospective multicenter observational study, we evaluated the real-world effectiveness and safety of Th2 biologic treatment in adults with CF with asthma and/or ABPA by comparing clinical outcomes before biologic and after biologic treatment. Overall, there was no significant improvement in ppFEV_1_ from baseline to 12 months after biologic treatment, but there was an improvement in the rate of ppFEV_1_ change 12 months before biologics vs after biologics. There was also no significant change in the average daily SCS dose or the frequency of PExs requiring hospitalization 12 months before biologics vs after biologics. Both Th2 biologics used (mepolizumab, benralizumab) were well tolerated, and no serious adverse effects were reported.

To date, and to our knowledge, only 1 case report and a small case series involving 3 adults have reported on their experience using Th2 biologics for the treatment of asthma/ABPA in the CF population.^17^^,^^18^ In the case report, mepolizumab use in an adult with CF with ABPA and asthma facilitated successful systemic steroid cessation, reduced inhaled corticosteroid dosing, and facilitated reexpansion of the left lower lobe on chest radiograph presumably related to reduced mucus plugging.^17^ The case series examined 3 adults with CF with asthma and suspected allergic fungal disease.^18^ After initiation of mepolizumab, there was a reduction in the requirement for oral corticosteroids but no change in the frequency of exacerbations. Two of the 3 individuals initiated tezacaftor-ivacaftor at the same time as mepolizumab, making it difficult to ascribe all the benefits to mepolizumab.

Case reports and case series can be prone to publication bias because positive findings are more likely to be reported and published.^22^ In this study, which reports the collective experience using Th2 biologics from 2 large CF centers in Canada, the clinical response to Th2 biologics was heterogeneous as demonstrated by the variable lung function responses and lack of significant improvement in most of the other outcomes of interest including exacerbation frequency and systemic steroid requirements. The airway inflammatory milieu in CF is complex; therefore, it is not unexpected that Th2 biologics may benefit all people with CF who meet the Th2 biologic eligibility criteria established for the population with asthma.^23^ Although individuals included in our study were required to have peripheral eosinophilia (> 300 cells/uL) or steroid-dependent disease, adherence to high-dose Inhaled corticosteroid (ICS)/Long-acting beta-agonist (LABA) combination therapy, and ≥ 2 exacerbations per year to be eligible for Th2 biologic reimbursement for severe eosinophilic asthma, these features are likely less specific as predictors of response in CF. In subgroup analyses, we observed a larger treatment response in individuals with asthma alone (vs combined with ABPA) and in individuals with higher peripheral eosinophil counts, suggesting that these might be predictors of Th2 biologic response in CF, but larger studies are required to confirm this.

Previous studies evaluating the safety of Th2 biologics in severe eosinophilic asthma have reported an acceptable adverse event profile in both the short and long term.^24^ In our study, adverse events were rare and did not result in treatment discontinuation.

There are several limitations of this observational study including the retrospective design and lack of a control group. Although to our knowledge, it represents the largest study evaluating Th2 biologics in pwCF to date, only 40 pwCF received Th2 biologics for asthma and/or ABPA combined during a follow-up period of 9 to 12 months. As a result, our analyses were likely underpowered to be conclusive regarding lack of a treatment effect, particularly for relatively infrequent outcomes (eg, PExs requiring hospitalization). The diagnosis of asthma and ABPA can be challenging in the CF population due to several overlapping clinical features with CF airways disease, which may have resulted in the inappropriate selection of patients for Th2 biologic therapy. However, the diagnoses of asthma and ABPA were reviewed by at least 2 experienced CF clinicians at each site reducing the risk of diagnostic misclassification. Based on our study inclusion criterion requiring the use of a Th2 biologic targeting eosinophilic inflammation for at least 3 months, we did not characterize the overall clinic populations with uncontrolled asthma and/or ABPA, which would have provided more context on the proportion treated with Th2 biologic therapy. This study exclusively involved adults with CF with moderate to severe airways disease; therefore, there may have been less opportunity to observe improvements in ppFEV_1_ given the potential for fixed airway obstruction. However, we did not observe improvements in other disease activity parameters including PExs requiring IV antibiotics or corticosteroid use. Finally, only a minority of the cohort was treated with highly effective modulator therapy (HEMT) (eg, ivacaftor, elexacaftor-tezacaftor-ivacaftor). Although this can be considered a strength because our results are less likely to be confounded by the introduction of HEMT during the observation period (unlike prior case reports and series), it also limits the generalizability of our study’s results because the clinical effectiveness of Th2 biologics may be different in a post-HEMT era and therefore warrants further evaluation. Furthermore, we were not able to evaluate whether pwCF maintained on biologic therapy before initiating HEMT can safely stop biologic therapy after HEMT initiation, and this important question also warrants further study to inform CF clinicians.

## Interpretation

Our results showed that Th2 biologics (mepolizumab and benralizumab specifically) were safe and improved the rate of change in lung function in CF adults with asthma or ABPA overlap. However, the responses were heterogeneous with respect to their impact on lung function improvement, PExs requiring hospitalization, and need for SCSs. Further study is needed to evaluate the role of Th2 biologics in the era of HEMT.

## Funding/Support

The authors have reported to *CHEST Pulmonary* that no funding was received for this study.

## Financial/Nonfinancial Disclosures

None declared.

## Supplementary Data

e-Online Data
