# Supplementary material for: A Multicenter Retrospective Study Evaluating IL-5-Targeted Biologic Therapies for the Treatment of Asthma and Allergic Bronchopulmonary Aspergillosis in Adults With Cystic Fibrosis
Source: CHEST Pulm. 2025 Mar 12;3(2):100163. doi: 10.1016/j.chpulm.2025.100163 (PMC13419108; doi:10.1016/j.chpulm.2025.100163)
Supplement: e-Online Data [file mmc1.docx]

**Supplementary Document**

**
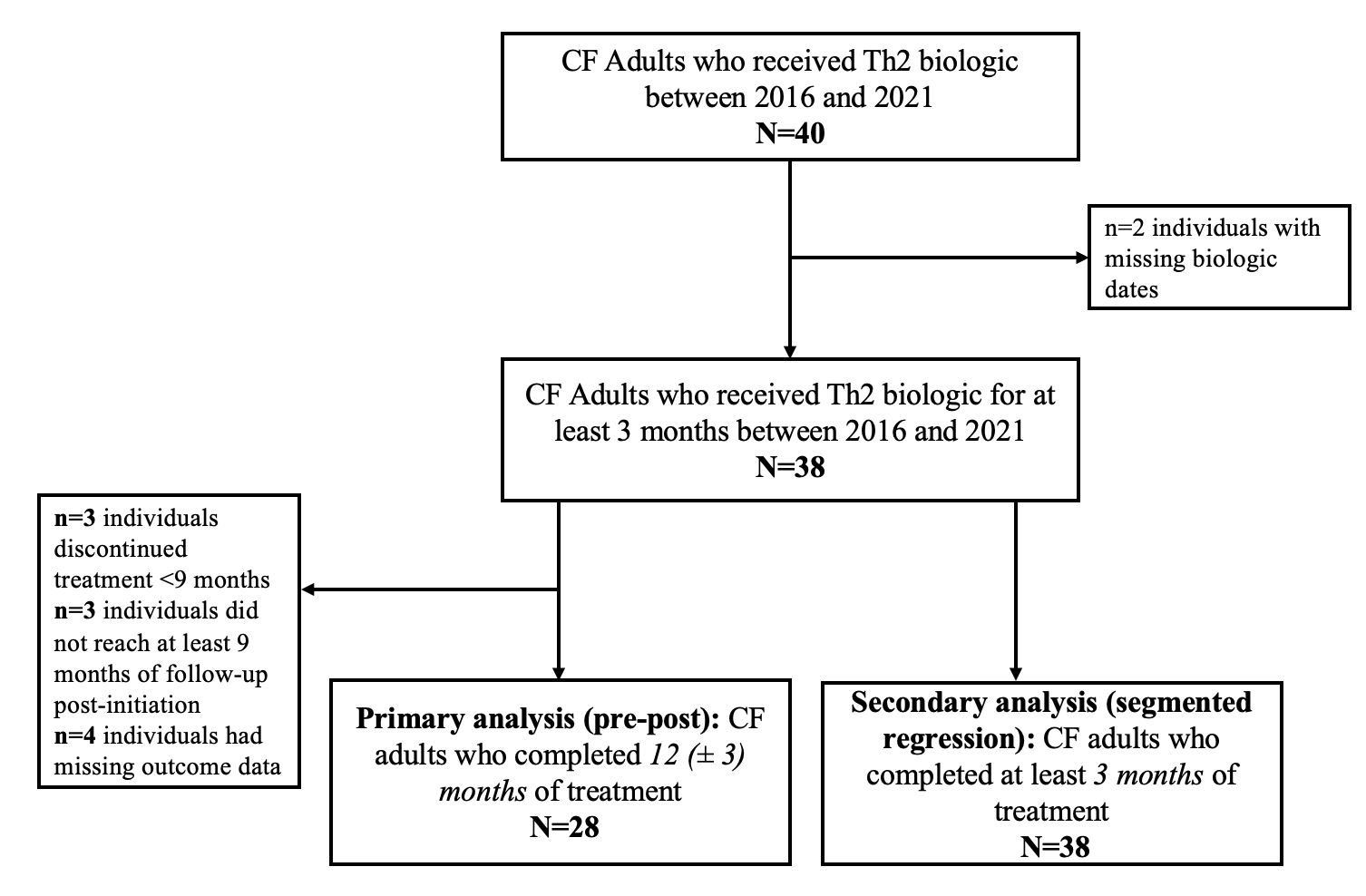
**

**Figure S1.** Flow diagram of creation of both analytic cohorts.


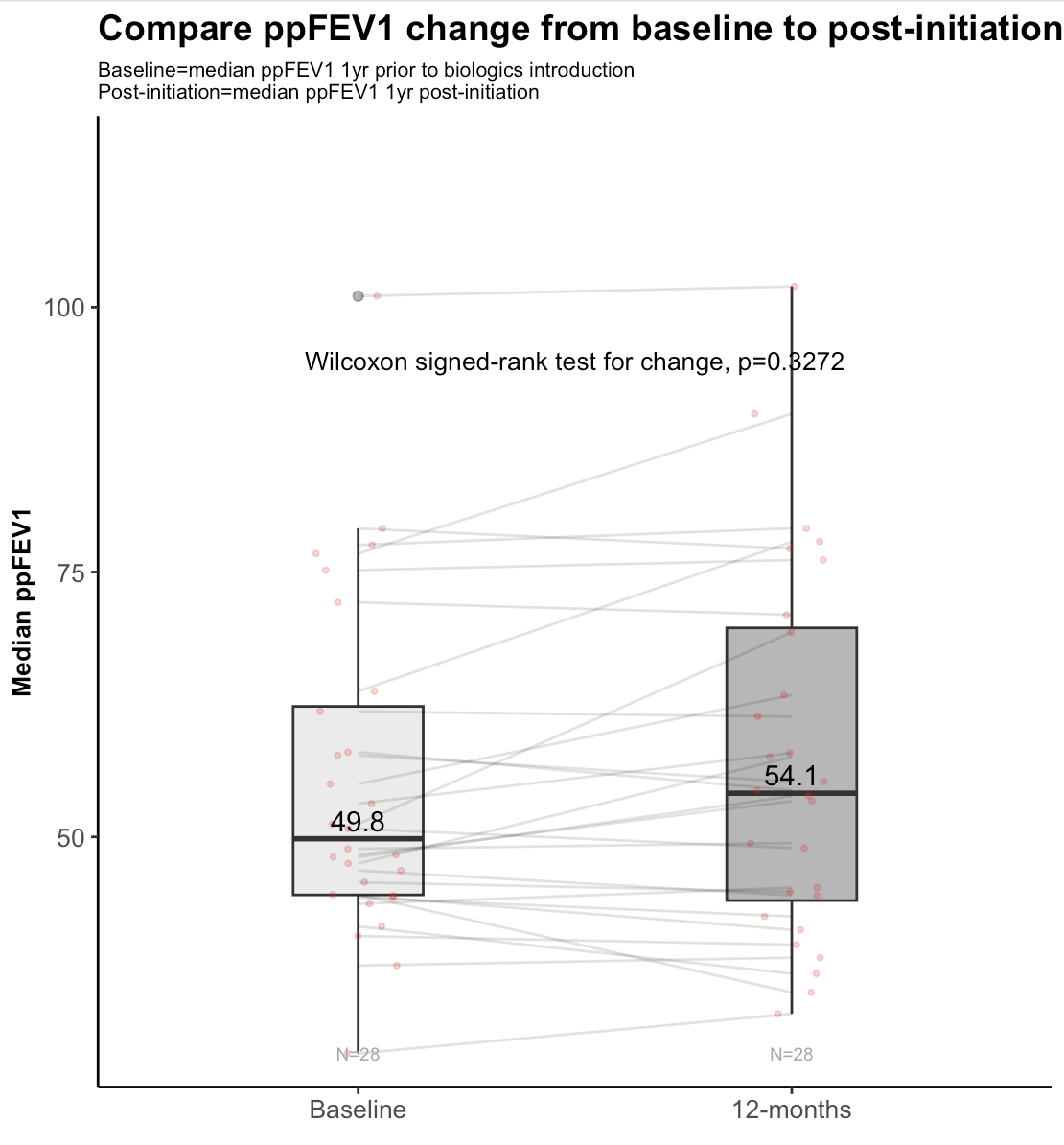


**Figure S2.** Comparison of the average (median) of ppFEV1 measurements from12 months prior to initiation of biologic treatment and 12 months post-biologic initiation. This sensitivity analysis reduces the average ppFEV1 at baseline by approximately 1% and decreases average ppFEV1 at 12 months by 0.4% and remains non-significant.

**
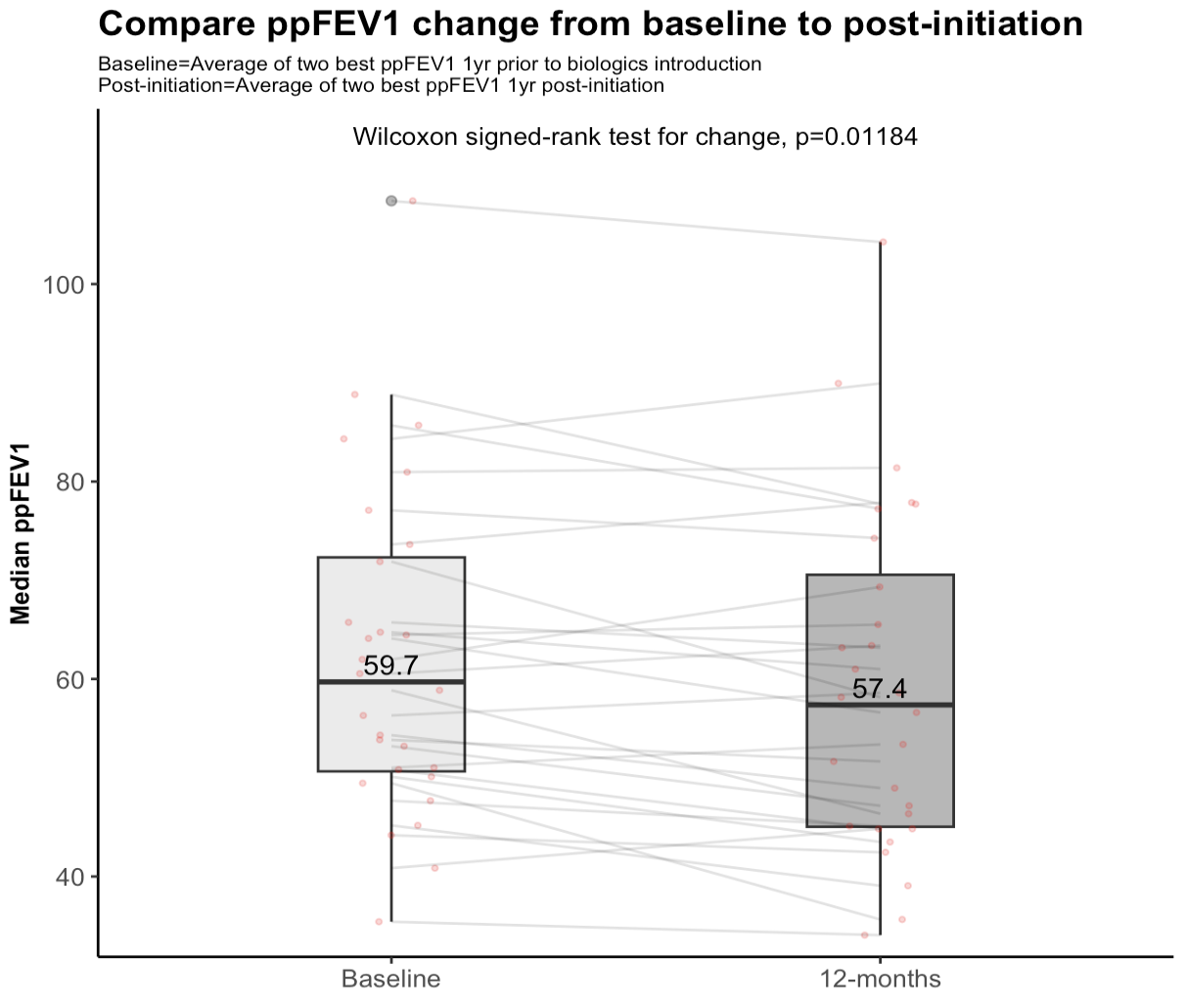
**

**Figure S3.** Taking the average (median) of the two best ppFEV1 measurements from 12 months prior to initiation of biologic treatment (baseline) and 12 months post-biologic. This sensitivity analysis increases the average ppFEV1 at baseline by 10% and increases 1 year average by 3% and results in statistically significant findings, but in the opposite direction. This approach likely compares a healthier pre-biologic status than the original analysis.

**
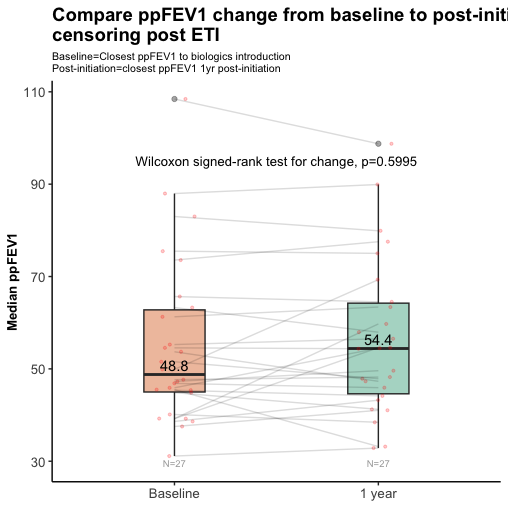
**

**Figure S4.** ppFEV1 change from baseline to 12 months post-biologic after excluding one individual who started a CFTR modulator post-biologic start

**
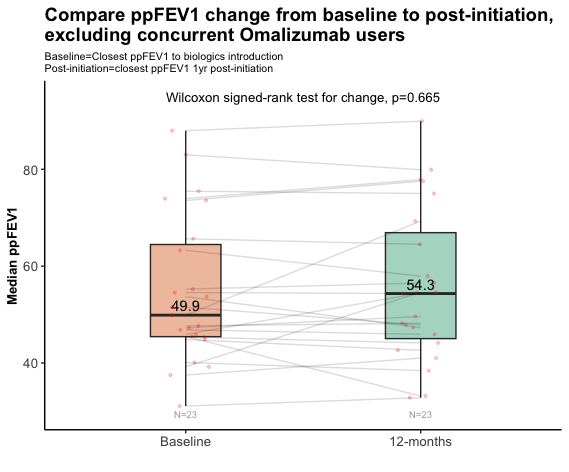
**

**
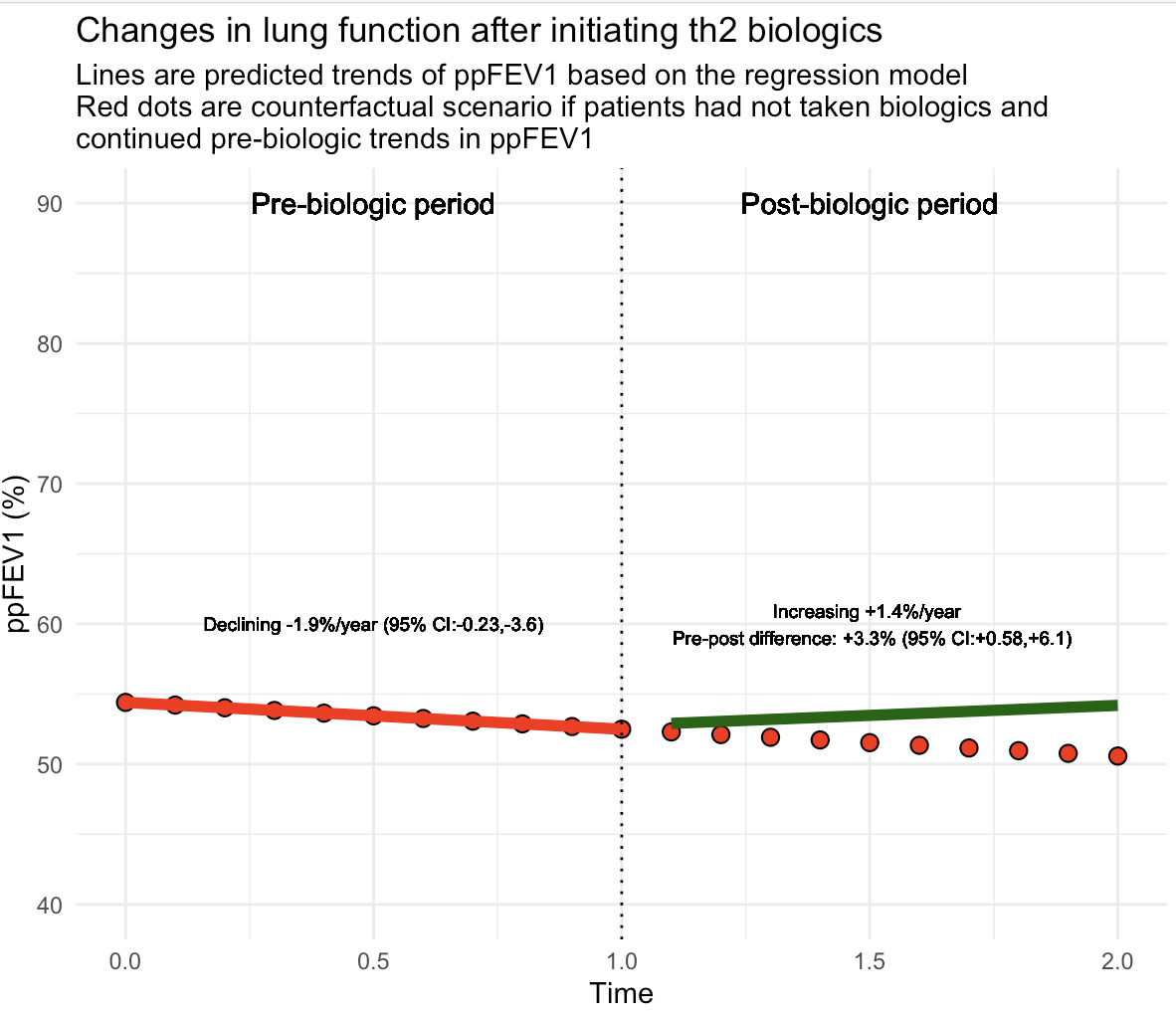
**

**Figure S5.** Removing concurrent omalizumab users (n=5) did not change primary findings.

**
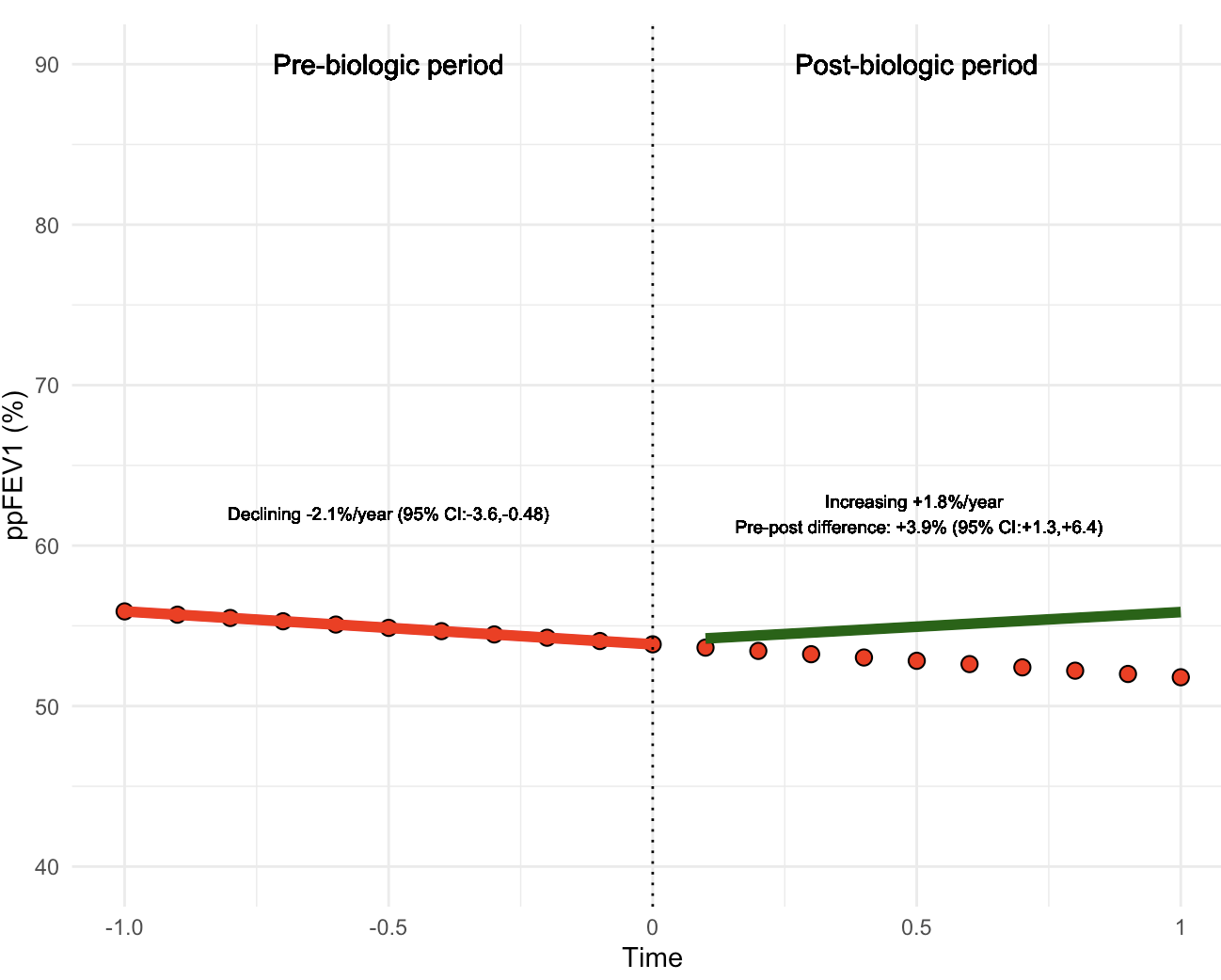
**

**Figure S6.** ppFEV1 change from 12 months pre- to 12 months post-biologic censoring individuals at modulator start


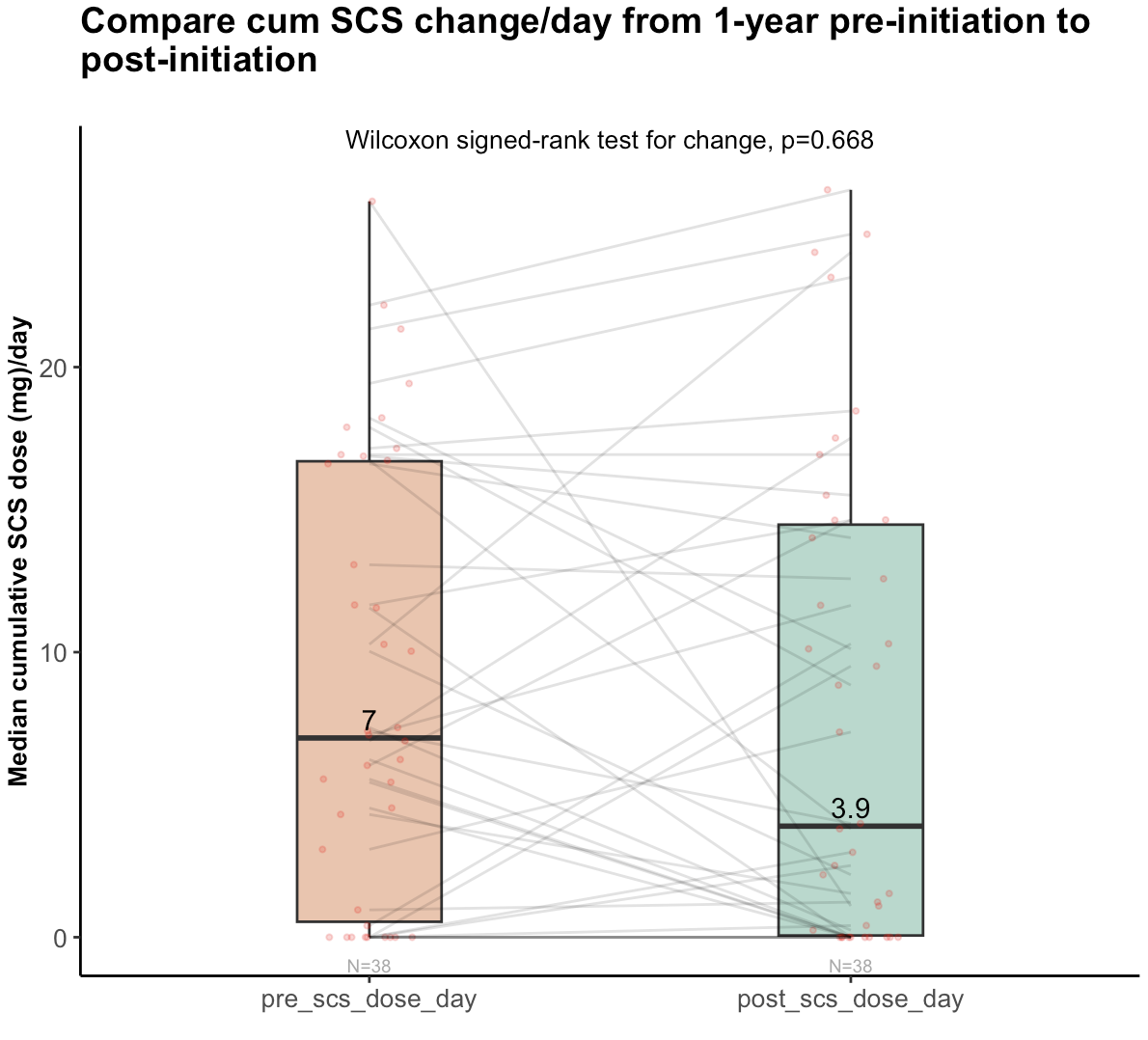


**Figure S7.** Median change in SCS dose mg/day from pre to post-initiation (p=0.668)

**
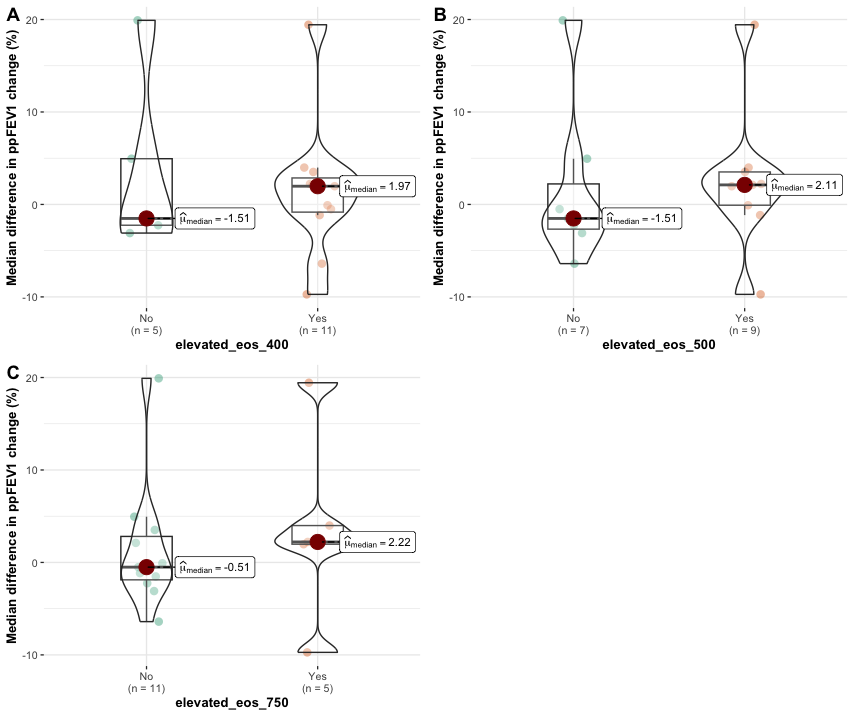
**

**Figure S8.** Median change in ppFEV1 among absolute eosinophil count (AEC) sub-groups not on systemic corticosteroids; (A) <400 vs. >=400 cells/μL (p=1.00); (B) <500 vs. >=500 cells/μL (p=0.408).

**
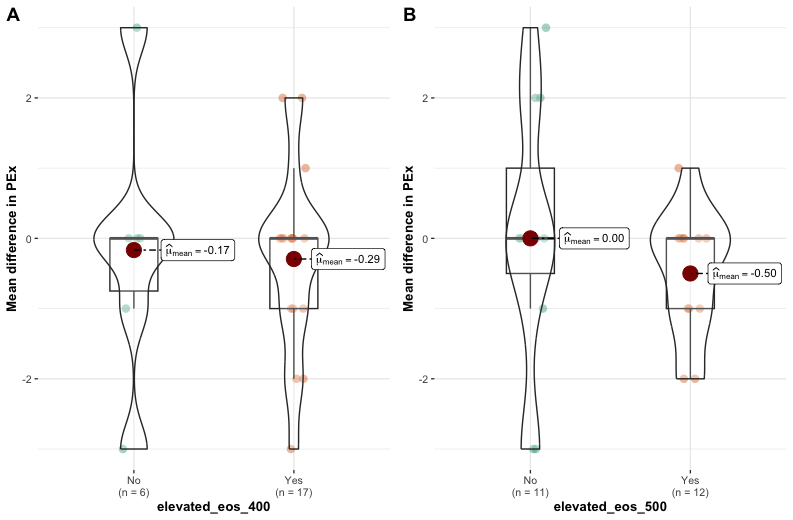
**

**Figure S9.** Mean change in PEx count among absolute eosinophil count (AEC) sub-groups not on systemic corticosteroids; (A) <400 vs. >=400 cells/μL (p=0.89); (B) <500 vs. >=500 cells/μL (p=0.44).


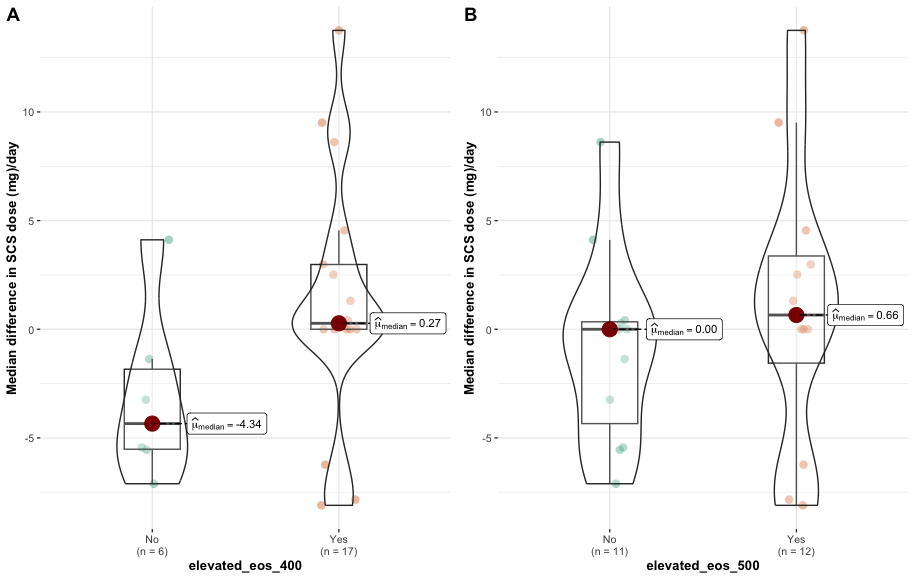


**Figure S10.** Median change in cumulative SCS dose (mg)/day among absolute eosinophil count (AEC) sub-groups not on systemic corticosteroids; (A) <400 vs. >=400 cells/μL (p=0.10); (B) <500 vs. >=500 cells/μL (p=0.48).

**
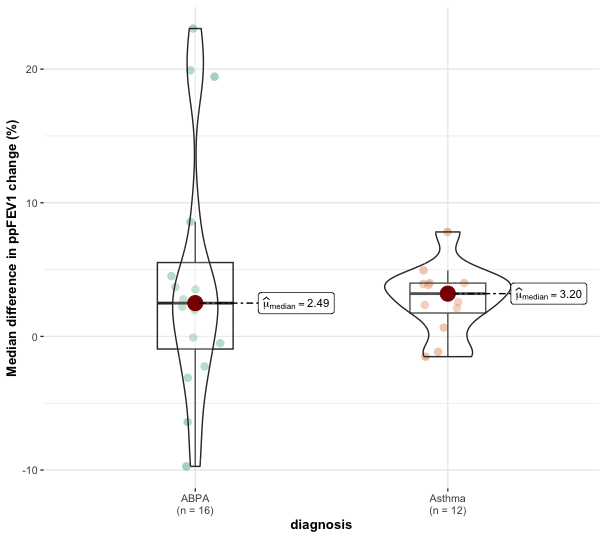
**

**Figure S11.** Median change in ppFEV1 among diagnostic sub-groups – asthma only vs. ABPA with or without asthma (p=0.69)


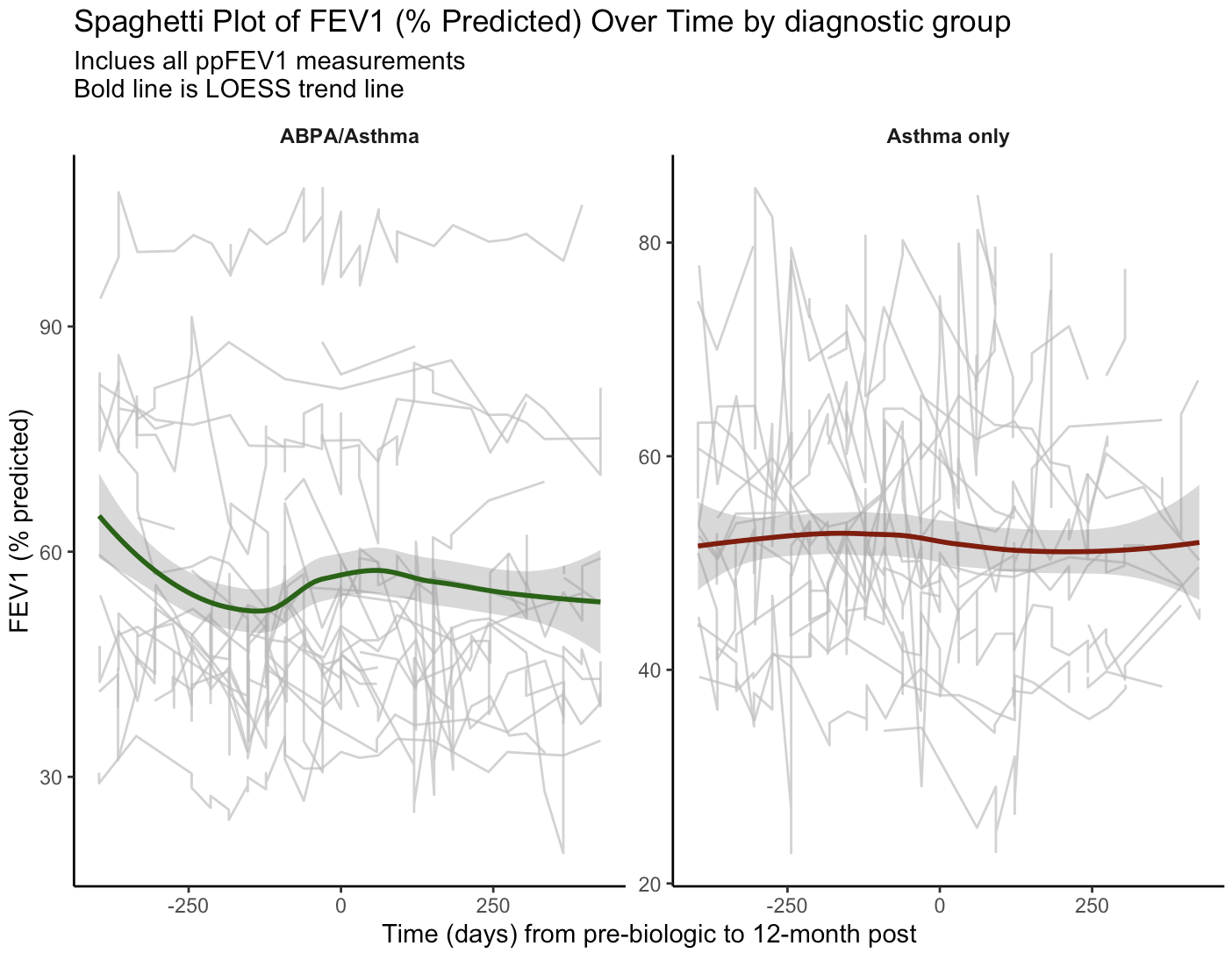


**Figure S12.** Spaghetti plots of Asthma and ABPA/Asthma patients. All ppFEV1 measurement 12 months prior and 12 months post included.

**
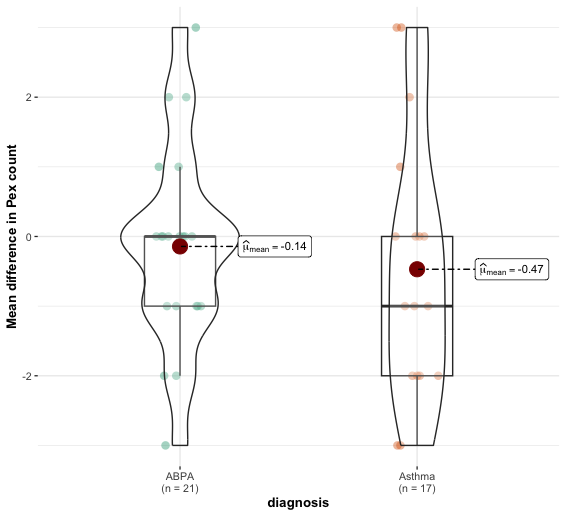
**

**Figure S13.** Mean change in PEx count among diagnostic sub-groups – asthma only vs. ABPA with or without asthma (p=0.56)


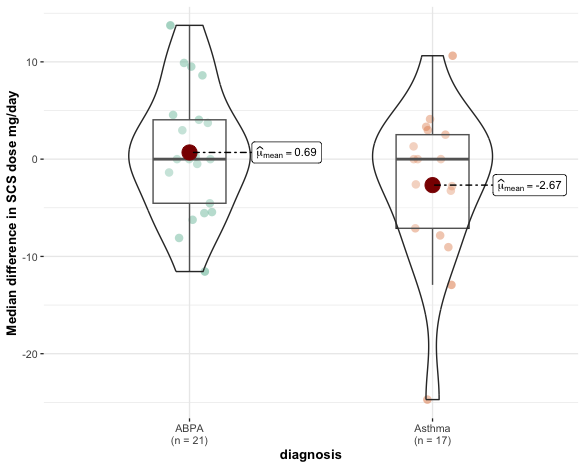


**Figure S14.** Median change in SCS dose mg/day among diagnostic sub-groups – asthma only vs. ABPA with or without asthma (p=0.17)

| **Characteristic** | **ABPA/Asthma**, N = 21^1^ | **Asthma only**, N = 17^1^ | **p-value**^2^ |
| --- | --- | --- | --- |
| **Age at biologic initiation (yrs)** | 33 (21, 60) | 35 (22, 67) | 0.7 |
| **Sex** |  |  | 0.10 |
| Female | 8 (38%) | 11 (65%) |  |
| Male | 13 (62%) | 6 (35%) |  |
| **CF genotype** |  |  | 0.5 |
| Hetero delF508 | 9 (43%) | 5 (29%) |  |
| Homo delF508 | 10 (48%) | 8 (47%) |  |
| Other | 2 (9.5%) | 4 (24%) |  |
| **Micro PA+** | 19 (90%) | 11 (65%) | 0.11 |
| **Micro Asp+** | 12 (57%) | 10 (59%) | >0.9 |
| **ppFEV1 at biologic initiation** | 50 (31, 105) | 48 (29, 79) | 0.7 |
| **BMI** | 22.8 (17.6, 27.4) | 22.8 (17.7, 33.6) | >0.9 |
| **Pancreatic status** |  |  | >0.9 |
| Insufficient | 18 (86%) | 15 (88%) |  |
| Sufficient | 3 (14%) | 2 (12%) |  |
| **CFRD** | 13 (62%) | 12 (71%) | 0.6 |
| **Total IgE (kU/L)** | 250 (2, 12,881) | 116 (2, 338) | 0.091 |
| **Elevated Aspergillus-specific IgE** | 10 (91%) | 4 (44%) | 0.08 |
| **Total WBC** | 13.6 (9.4, 22.6) | 14.2 (7.3, 28.8) | 0.7 |
| **Eosinophils (cells/L)** | 0.45 (0.10, 2.49) | 0.60 (0.19, 1.45) | 0.6 |
| **Elevated Eosinophils** | 9 (43%) | 10 (59%) | 0.3 |
| **Systemic corticosteroid use prior to biologic initiation** | 16 (76%) | 13 (76%) | >0.9 |
| **No. of pre-biologic FEV1 measurements** | 16 (8, 24) | 16 (5, 29) | 0.8 |
| **No. of post-biologic FEV1 measurements** | 12 (4, 34) | 13 (6, 26) | 0.7 |
| **Follow-up time post-biologics (yrs)** | 1.08 (0.34, 1.17) | 1.00 (0.25, 1.17) | 0.3 |
| **Transplant** | 1 (4.8%) | 0 (0%) | >0.9 |
| **Deaths** | 0 (0%) | 2 (12%) | 0.2 |

**Table S1.** Clinical characteristics of CF patients receiving Th2 biologics stratified by diagnosis type

**Abbreviations:** ABPA, allergic bronchopulmonary aspergillosis; Asp, aspergillus; BMI, body mass index; CFRD, CF-related diabetes; PA, Pseudomonas aeruginosa; ppFEV1, percent-predicted FEV1

| **Characteristic** | **Non-responder**, N = 20^1^ | **Responder**, N = 8^1^ | **p-value**^2^ |
| --- | --- | --- | --- |
| **Age at biologic initiation (yrs)** | 38 (31, 57) | 36 (23, 41) | 0.2 |
| **Sex** |  |  | 0.7 |
| Female | 9 (45%) | 5 (63%) |  |
| Male | 11 (55%) | 3 (38%) |  |
| **CF genotype** |  |  | 0.4 |
| Hetero delF508 | 6 (30%) | 5 (63%) |  |
| Homo delF508 | 9 (45%) | 2 (25%) |  |
| Other | 5 (25%) | 1 (13%) |  |
| **Diagnosis** |  |  | >0.9 |
| ABPA | 2 (10%) | 1 (13%) |  |
| Asthma | 9 (45%) | 3 (38%) |  |
| Both | 9 (45%) | 4 (50%) |  |
| **Micro PA+** | 15 (75%) | 8 (100%) | 0.3 |
| **Micro Asp+** | 15 (75%) | 5 (63%) | 0.7 |
| **ppFEV1 at biologic initiation** | 51 (45, 66) | 44 (37, 56) | 0.3 |
| **BMI** | 23.6 (21.1, 26.4) | 21.9 (19.1, 24.9) | 0.4 |
| **Pancreatic status** |  |  | >0.9 |
| Insufficient | 16 (80%) | 7 (88%) |  |
| Sufficient | 4 (20%) | 1 (13%) |  |
| **CFRD** | 13 (65%) | 6 (75%) | >0.9 |
| **Total IgE (kU/L)** | 127 (50, 255) | 242 (52, 363) | 0.4 |
| **Aspergillus-specific Precipitins** |  |  | >0.9 |
| negative | 12 (80%) | 3 (75%) |  |
| positive | 3 (20%) | 1 (25%) |  |
| **Elevated Aspergillus-specific IgE** | 6 (67%) | 4 (80%) | >0.9 |
| **Total WBC** | 14.5 (11.5, 17.1) | 16.4 (12.5, 18.3) | 0.6 |
| **Eosinophils (cells/L)** | 0.53 (0.36, 0.76) | 0.65 (0.35, 1.07) | 0.5 |
| **Elevated Eosinophils** | 10 (50%) | 4 (50%) | >0.9 |
| ^1^Median (IQR); n (%) | | | |
| ^2^Wilcoxon rank sum test; Fisher's exact test; Wilcoxon rank sum exact test | | | |

**Table S2.** Clinical characteristics of CF patients receiving Th2 biologics stratified by responder group. Response was defined as >=3% improvement in ppFEV1 among individuals who completed 12 (± 3) months of Th2 biologic treatment

**Abbreviations:** ABPA, allergic bronchopulmonary aspergillosis; Asp, aspergillus; BMI, body mass index; CFRD, CF-related diabetes; PA, Pseudomonas aeruginosa; ppFEV1, percent-predicted FEV1


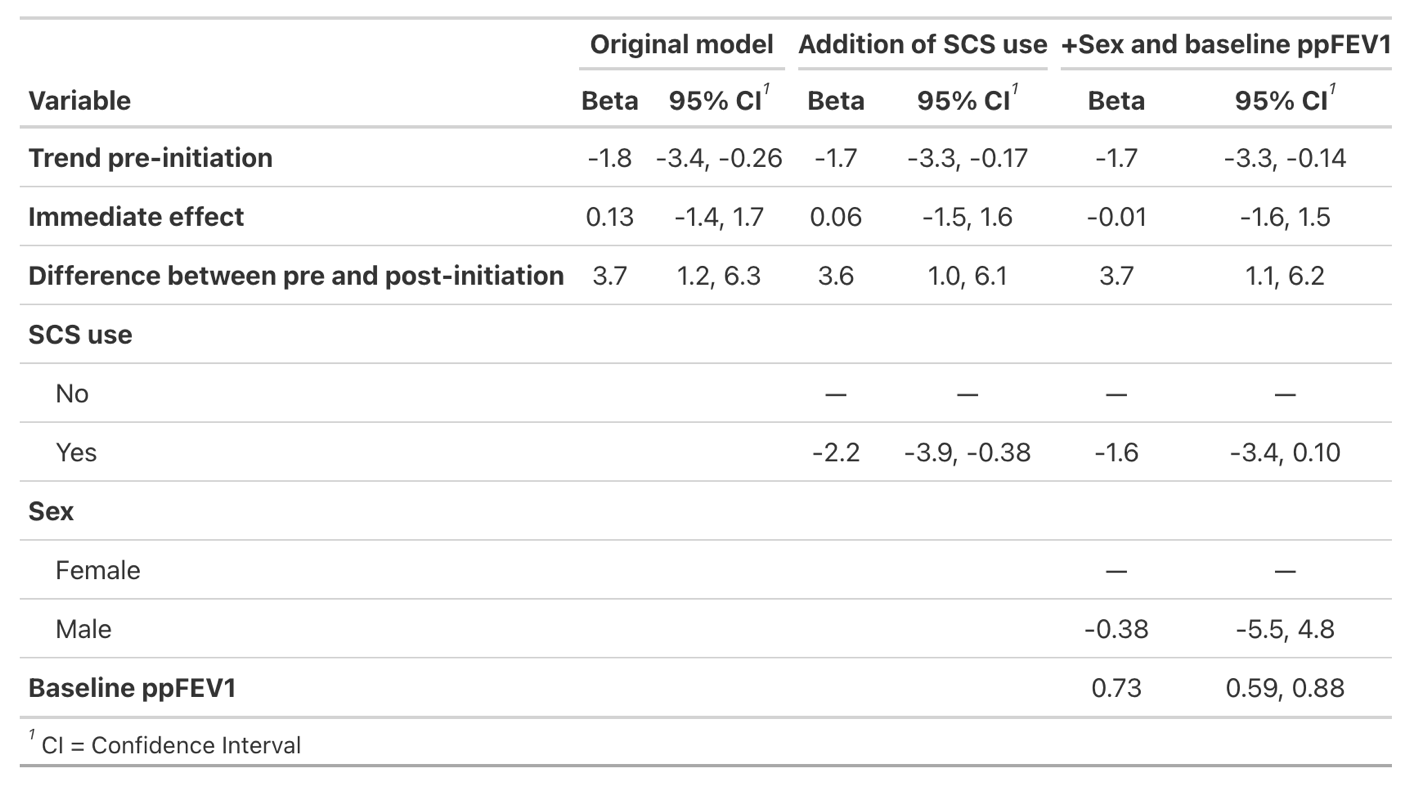


**Table S3.** Comparison of segmented regression results and addition of covariates in model.
